# Combined bulk RNA and single-cell RNA analyses reveal TXNL4A as a new biomarker for hepatocellular carcinoma

**DOI:** 10.3389/fonc.2023.1202732

**Published:** 2023-05-25

**Authors:** Yifan Li, Qiaozhen Zhu, Shuchang Zhou, Jiangtao Chen, Aoyu Du, Changjiang Qin

**Affiliations:** ^1^Department of Gastrointestinal Surgery, Huaihe Hospital of Henan University, Kaifeng, Henan, China; ^2^Infection and Immunity Institute and Translational Medical Center, Huaihe Hospital, Kaifeng, Henan, China; ^3^Department of Plastic Surgery, Huaihe Hospital of Henan University, Kaifeng, Henan, China

**Keywords:** RNA splicing pathway, TXNL4A, hepatocellular carcinoma, tumor immune, single-cell RNA

## Abstract

**Introduction:**

Hepatocellular carcinoma (HCC) has a high mortality rate worldwide. The dysregulation of RNA splicing is a major event leading to the occurrence, progression, and drug resistance of cancer. Therefore, it is important to identify new biomarkers of HCC from the RNA splicing pathway.

**Methods:**

We performed the differential expression and prognostic analyses of RNA splicing-related genes (RRGs) using The Cancer Genome Atlas-liver hepatocellular carcinoma (LIHC). The International Cancer Genome Consortium (ICGC)-LIHC dataset was used to construct and validate prognostic models, and the PubMed database was used to explore genes in the models to identify new markers. The screened genes were subjected to genomic analyses, including differential, prognostic, enrichment, and immunocorrelation analyses. Single-cell RNA (scRNA) data were used to further validate the immunogenetic relationship.

**Results:**

Of 215 RRGs, we identified 75 differentially expressed prognosis-related genes, and a prognostic model incorporating thioredoxin like 4A (TXNL4A) was identified using least absolute shrinkage and selection operator regression analysis. ICGC-LIHC was used as a validation dataset to confirm the validity of the model. PubMed failed to retrieve HCC-related studies on TXNL4A. TXNL4A was highly expressed in most tumors and was associated with HCC survival. Chi-squared analyses indicated that TXNL4A expression positively correlated positively with the clinical features of HCC. Multivariate analyses revealed that high TXNL4A expression was an independent risk factor for HCC. Immunocorrelation and scRNA data analyses indicated that TXNL4A was correlated with CD8 T cell infiltration in HCC.

**Conclusion:**

Therefore, we identified a prognostic and immune-related marker for HCC from the RNA splicing pathway.

## Introduction

Hepatocellular carcinoma (HCC) is among the causes of high morbidity and mortality rates because it manifests with high heterogeneity and early diagnosis is difficult (1, 2). Surgery and targeted therapy can treat certain patients with HCC; however, many patients present with metastases, postoperative recurrence, and chemoresistance, making prognosis unsatisfactory for them (3, 4). Therefore, the search for HCC biomarkers has important implications in its diagnosis and treatment.

Abnormal gene expression results in abnormal RNA splicing, resulting in disease development (5–7). Studies on genetic and functional data report that RNA splicing factors can affect the tumor process, suggesting that RNA splicing is a crucial target for cancer therapy (8–10). Moreover, abnormal RNA splicing can promote antiapoptotic activity and protect tumor cells from chemotherapy drugs (11). Aberrant RNA shearing can also affect the regulation of tumor immunity (12).

Single-cell RNA (scRNA) sequencing can be used to comprehensively analyze tissue heterogeneity (13). Recently, scRNA sequencing importantly contributed to improving the resolution of identifying the unique characteristics of each cell (13). scRNA sequencing can effectively analyze tumor immune infiltration (14). Therefore, we used scRNA analyses in this study to evaluate the genetic abundance in HCC immune cells.

We aimed to identify a novel biomarker for HCC from the RNA splicing pathway, which could help explore the potential mechanisms underlying the effects of abnormal RNA splicing on HCC.

## Data and methods

### Data acquisition

A total of 215 RNA splicing-related genes (RRGs) were obtained from Genecards. Gene expression profiles for The Cancer Genome Atlas (TCGA)-liver hepatocellular carcinoma (LIHC) (cancer = 374; normal = 50) and the corresponding clinical data were obtained from Genomic Data Commons TCGA (https://portal.gdc.cancer.gov/). GSE64041 (cancer = 60; normal = 60) and GSE39791 (cancer = 72; normal = 72) were obtained from the Gene Expression Omnibus database (https://www.ncbi.nlm.nih.gov/geo/). The International Cancer Genome Consortium (ICGC)-HCC-Japan (cancer = 241) procured patient gene expression data from the ICGC database (https://dcc.icgc.org/) as well as clinical information. Other databases used in this study included Gene Expression Profiling Interactive Analysis (GEPIA) (http://gepia.cancer-pku.cn/detail.php?clicktag=matrix), The University of Alabama at Birmingham Cancer data analysis Portal (UALCAN) (https://ualcan.path.uab.edu/index.html), Tumor Immune Estimator Resource (TIMER) https://cistrome.shinyapps.io/timer/), and Kaplan–Meier Plotter (https://kmplot.com/analysis/). The single-cell datasets GSE140228 and GSE166635 for HCC were obtained from the Tumor Immune Single Cell Hub 2 (TISCH2) database (http://tisch.comp-genomics.org/home/). Data analysis and visualization tools included R software (version 4.3.2) and the Xiantao tool (https://www.xiantao.love/).

### Genetic screening of RRGs for a novel HCC biomarker

First, R was used to analyze the differential expression and prognosis of RRGs. Subsequently, it was used to analyze differential prognosis-related RRGs (|logFC| > 1; hazard ratio [HR] > 1; p < 0.05). The risk model was constructed using least absolute shrinkage and selection operator (Lasso) regression analysis, and studies on genes related to HCC were searched using the PubMed database.

### Expression of thioredoxin-like 4A in HCC

We conducted pan-cancer analyses using the TIMER database to identify the expression of TXNL4A. Differences in the expression of TXNL4A in normal and HCC tissues in TCGA-LIHC were analyzed using the R packages and validated in the same way as in the GSE64041 and GSE39791 datasets. The UALCAN and GEPIA online databases were further used to analyze differences in biomarker expression.

### Analysis of TXNL4A and its clinicopathological correlation with survival

R was used to analyze the relationship between TXNL4A expression in TCGA-LIHC and ICGC and overall HCC survival. Furthermore, GEPIA and UALCAN databases were used to analyze the correlation between biomarkers and HCC survival and to plot Kaplan–Meier curves. Using clinical data from TCGA-LIHC, Cardinal and Fisher tests were performed to identify the relationship between TXNL4A and clinical characteristics (TNM stage, pathologic stage, histologic grade, vascular invasion, and overall survival [OS]).

### TXNL4A as an independent prognostic factor

We performed univariate and multivariate Cox regression analyses to identify independent prognostic factors for HCC and to determine the prognostic impact of TXNL4A and clinical characteristics.

### Functional enrichment analyses of biomarkers

To further explore the biological role of biomarker expression in HCC, TCGA-LIHC samples were divided into high- and low-TXNL4A groups based on median TXNL4A expression, and gene set enrichment analysis (GSEA) was performed using all differentially expressed genes (DEGs) between groups. For this step, the GSEA software (version 4.2.3) with the parameters “c2.all.v2022.1.Hs.symbols.gmt,” “500 times,” and “no collapse” were used.

### Relationship between TXNL4A and HCC immune infiltration

Because RNA splicing plays a crucial role in cancer immunity (15, 16), the relationship between the expression of TXNL4A and HCC immune infiltration was analyzed using TIMER database and validated using the CIBERSORT algorithm along with the gene set cancer analysis (GSCA) database.

### Analysis of single-cell sequencing RNA material

The scRNA sequencing technology is an effective tool for analyzing immune infiltration (17–19) Therefore, two scRNA sequencing datasets (GSE140228_10X and GSE166635) were analyzed to investigate TXNL4A expression in each cell cluster using the TISCH2 database (20).

### Statistical analyses

All statistical analyses in this study (Wilcoxon’s rank sum test, t-test, Chi-squared test, and Fisher’s test) were conducted using R software and the Xiantao tool. P-values were two-tailed, and p < 0.05 was considered statistically significant.

## Results

### TXNL4A was screened as a new biomarker for HCC

**Figure 1:**
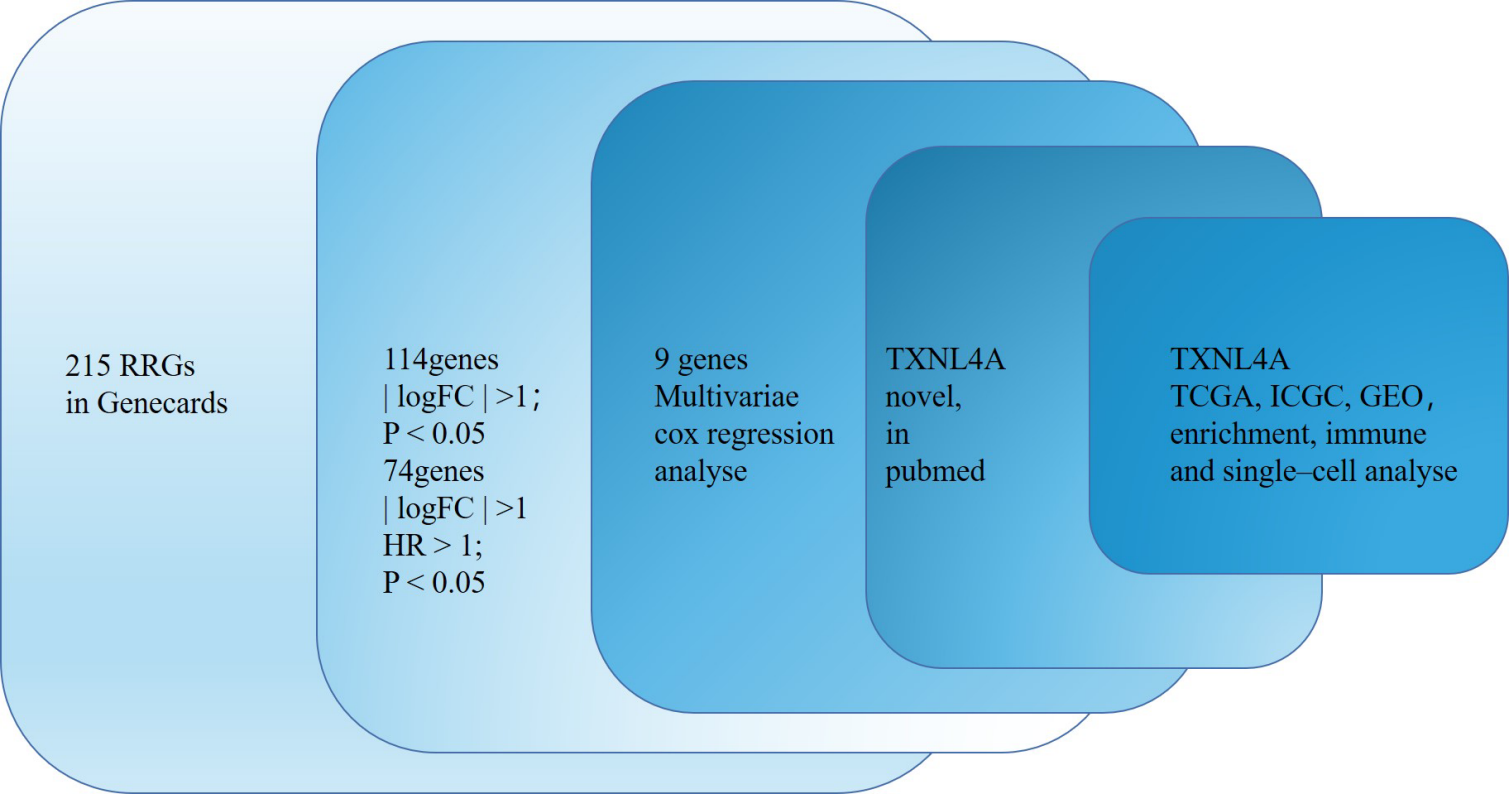
Flow chart of the study.

**Figure 2:**
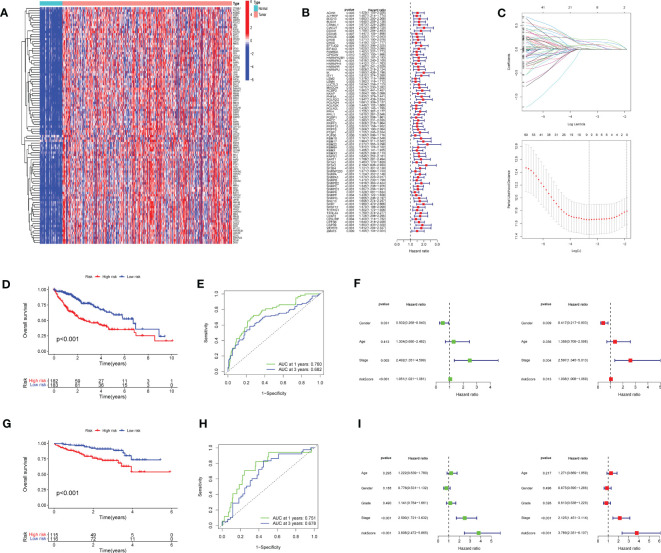
Prognostic model for hepatocellular carcinoma (HCC): **(A)** Heat map of differentially expressed genes (DEGs) **(B)** 74 DEGs exhibiting unfavorable prognoses for HCC **(C)** Lasso Cox regression model plotting partial likelihood deviation versus log(λ) **(D)** High-risk rating score resulting in poor overall survival **(E)** Model exhibiting good predictive performance **(F)** Risk scores as independent risk factors **(G–I)** International Cancer Genome Consortium dataset validates the scoring model.

The flow chart of this study is shown in **Figure 1**. Of 215 RRGs, we identified 114 DEGs. Of these DEGS, we identified 74 PRGs (**Figure 2A**). Thereafter, 100 differential prognosis-related genes were obtained by integrating the previous results (**Figure 2B**). An HCC risk model with nine RRGs (NCBP2, PHF5A, POLR2L RBM17, RBM22, SF3A3, SF3B4, TXNL4A, and UPF3B) was obtained using Lasso regression analyses. We also analyzed the plausibility of the model (**Figures 2C–I**). The list of RRGs, gene coefficients, a scoring formula, and risk group details are provided in the **Supplementary Material**. The PubMed database was searched for articles related to genes and HCC, and the results revealed that TXNL4A might be a new biomarker for HCC.

### Expression of TXNL4A in HCC

**Figure 3:**
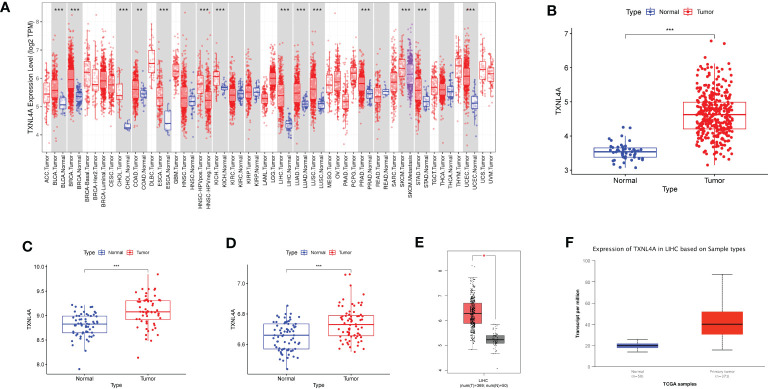
Differences in the expression of TXNL4A among different datasets: **(A)** Tumor Immune Estimator Resource database reveals the expression landscape of TXNL4A in pan-cancer analyses **(B)** The Cancer Genome Atlas-Liver Hepatocellular Carcinoma revealed high TXNL4A expression in cancer tissues **(C)** TXNL4A expression in the GSE64041 dataset **(D)** Differential expression of TXNL4A in the GSE39791 dataset **(E)** Validation of differential expression in the Gene Expression Profiling Interactive Analysis database **(F)** Validation of differential expression in the University of Alabama at Birmingham Cancer data analysis Portal database. *p < 0.05; **p < 0.01; ***p < 0.001.

The results of the TIMER database differential analyses indicated that TXNL4A was highly expressed in HCC, BLCA, BRCA, CHOL, COAD, ESCA, KICH, LUAD, LUSC, PRAD, and UCEC datasets (**Figure 3A**). The analyses of differential gene expression in HCC using R resulted in high TXNL4A expression in the tumor samples (**Figure 3B**) The same results were replicated in the GSE64041 and GSE39791 datasets (**Figures 3C, D**). GEPIA and UALCAN databases were used for further validation, and the difference in TXNL4A expression remained significant (**Figures 3E, F**).

### Survival analyses

**Figure 4:**
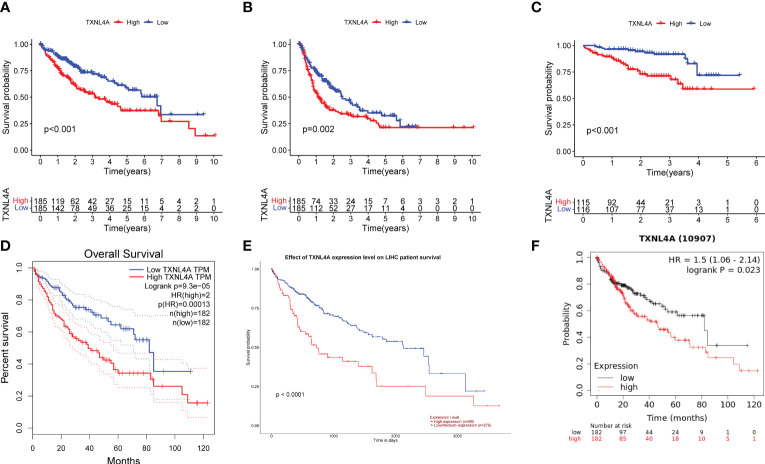
Effect of TXNL4A on the overall survival (OS) of patients with hepatocellular carcinoma (HCC): **(A)** In The Cancer Genome Atlas-Liver Hepatocellular Carcinoma, high TXNL4A expression resulted in a poorer OS **(B)** High TXNL4A expression resulted in a poor disease-free survival **(C)** Effect of TXNL4A on the OS of International Cancer Genome Consortium samples **(D)** Validation of differential expression in the Gene Expression Profiling Interactive Analysis database **(E)** Validation of differential expression in the University of Alabama at Birmingham Cancer data analysis Portal database **(F)** Effect of Kaplan–Meier plotter to validate the effect of TXNL4A on OS of patients with HCC.

The relationship between TXNL4A and HCC with regard to OS and progression-free interval (PFI) was analyzed using log-rank tests, and all survival analyses revealed that patients with high HCC expression exhibited worse survival than those with low HCC expression (**Figures 4A, B**) (OS: p < 0.001; PFI: p = 0.002). The ICGC data revealed that patients with low TXNL4A expression had better OS rates (**Figure 4C**) (p < 0.001). The effects of TXNL4A on the survival of patients with HCC was further validated by analyzing the results on the GEPIA, UALCAN, and Kaplan–Meier plotter databases. All database results revealed that patients with high TXNL4A expression experienced worse prognosis than those with low TXNL4A expression (**Figures 4D–F**) (GEPIA: p = 0.00013; UALCAN: p < 0.0001; Kaplan–Meier: p = 0.023).

### Correlation analyses between TXNL4A expression and clinical characteristics

Results of the Chi-squared test revealed that TXNL4A expression correlated with grade (p < 0.001), T stage (p < 0.001), pathologic stage (p<0.001), levels of serum alpha-fetoprotein (AFP) (p = 0.006), vascular invasion (p = 0.022), OS (p < 0.001), and Disease free survival (p = 0.022). TXNL4A expression showed no correlation with the N-stage (p = 0.648) or M-stage (p = 0.523). The detailed results of all analyses are shown in **Table 1**.

**Table 1:** The relationship between TXNL4A and clinical features of hepatocellular carcinoma.

Characteristic	Low-TXNL4A	High-TXNL4A	p	statistic	method
n	187	187			
T stage, n (%)			< 0.001	19.11	Chisq.test
T1	111 (29.9%)	72 (19.4%)			
T2	35 (9.4%)	60 (16.2%)			
T3	37 (10%)	43 (11.6%)			
T4	3 (0.8%)	10 (2.7%)			
N stage, n (%)			0.648	0.21	Chisq.test
N0	125 (48.4%)	129 (50%)			
N1	1 (0.4%)	3 (1.2%)			
M stage, n (%)			0.523	0.41	Chisq.test
M0	124 (45.6%)	144 (52.9%)			
M1	3 (1.1%)	1 (0.4%)			
Pathologic stage, n (%)			< 0.001	16.41	Chisq.test
Stage I	105 (30%)	68 (19.4%)			
Stage II	34 (9.7%)	53 (15.1%)			
Stage III	35 (10%)	50 (14.3%)			
Stage IV	4 (1.1%)	1 (0.3%)			
Histologic grade, n (%)			< 0.001	17.12	Chisq.test
G1	38 (10.3%)	17 (4.6%)			
G2	94 (25.5%)	84 (22.8%)			
G3	52 (14.1%)	72 (19.5%)			
G4	2 (0.5%)	10 (2.7%)			
Vascular invasion, n (%)			0.022	5.21	Chisq.test
No	122 (38.4%)	86 (27%)			
Yes	49 (15.4%)	61 (19.2%)			
OS event, n (%)			< 0.001	11.33	Chisq.test
Alive	138 (36.9%)	106 (28.3%)			
Dead	49 (13.1%)	81 (21.7%)			

### TXNL4A as an independent risk factor for HCC

To further explore the effect of TXNL4A on HCC, we performed univariate and multivariate Cox regression analyses using R. The results revealed that TXNL4A could be an independent prognostic factor for HCC (univariate: HR (95% confidence interval [CI]): 1.947 (1.364–2.780), p < 0.001; multivariate: HR (95% CI): 1.833 (1.130–2.976), p = 0.014) (**Table 2**).

**Table 2:** TXNL4A is an independent prognostic factor for hepatocellular carcinoma.

Characteristics	Total(N)	HR(95% CI) Univariate analysis	P Univariate	HR(95% CI) Multivariate analysis	P Multivariate
T stage	370				
T1&T2	277				
T3&T4	93	2.598 (1.826-3.697)	<0.001	1.724 (0.234-12.723)	0.593
N stage	258				
N0	254				
N1	4	2.029 (0.497-8.281)	0.324		
M stage	272				
M0	268				
M1	4	4.077 (1.281-12.973)	0.017	1.342 (0.319-5.644)	0.688
Pathologic stage	349				
Stage I & Stage II	259				
Stage III & Stage IV	90	2.504 (1.727-3.631)	<0.001	1.332 (0.181-9.805)	0.778
Tumor status	354				
Tumor free	202				
With tumor	152	2.317 (1.590-3.376)	<0.001	1.830 (1.141-2.937)	0.012
Gender	373				
Female	121				
Male	252	0.793 (0.557-1.130)	0.200		
Histologic grade	368				
G1 & G2	233				
G3 & G4	135	1.091 (0.761-1.564)	0.636		
TXNL4A	373				
Low	187				
High	186	1.947 (1.364-2.780)	<0.001	1.833 (1.130-2.976)	0.014

### Enrichment analysis of TXNL4A

**Figure 5:**
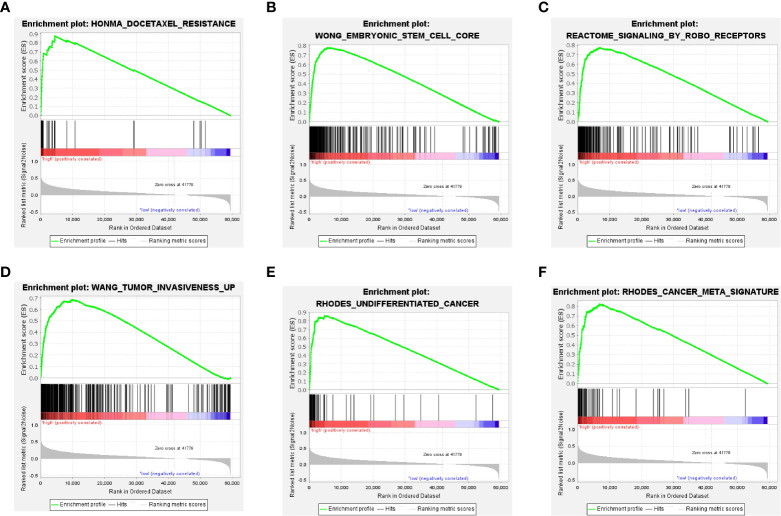
Pathways of DEGs in the high-TXNL4A group **(A)** Docetaxel resistance-related pathway **(B)** Embryonic stem cell-related pathway **(C)** ROBO receptor-related pathway **(D, E)** Tumor invasion-related pathway **(F)** Tumor-related pathway.

We unveiled the DEG pathways in six groups with high TXNL4A expression. The results revealed that the group with high TXNL4A expression exhibited increased docetaxel tolerance, embryonic stem cells, ROBO receptor signaling pathway dysregulation, tumor invasion, undifferentiated carcinoma, DNA damage, replication, and dysregulation of other pathways (**Figures 5A–F**).

### Relationship between TXNL4A and tumor immunity

**Figure 6:**
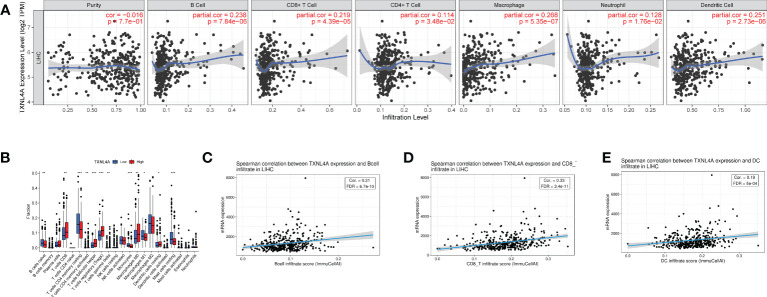
The immune analysis: **(A)** TIMER: TXNL4A correlated with tumor immune infiltration **(B)** CIBERSORT: reveals a close relationship between TXNL4A and CD8 T cells **(C–E)** Gene Set Cancer Analysis: TXNL4A expression positively correlates with the degree of infiltration of B cells, CD8 T cells, natural killer cells, and dendritic cells.

TIMER database analyses revealed that TXNL4A expression in HCC was positively correlated with the levels of B cells, CD4 T cells, CD8 T cells, macrophages, neutrophils, and dendritic cells (DCs) (**Figure 6A**). CIBERSORT algorithm analyses revealed high levels of CD8 cells, regulatory T cells, and macrophages M0 in the group with high TXNL4A expression (**Figure 6B**). The GSCA database revealed that TXNL4A expression was positively correlated with levels of B cells, CD8 T cells, and DCs (**Figure 6C**) Therefore, TXNL4A may be immunologically correlated with HCC, especially with levels of CD8 T cells.

### Single-cell sequencing data analyses

**Figure 7:**
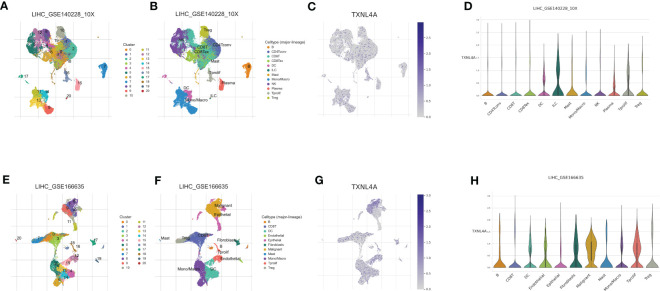
TXNL4A in different cell clusters: **(A)** GSE140228 was identified as 21 cell clusters **(B)** Cell cluster annotation of GSE140228 **(C)** Distribution of TXNL4A abundance in immune cell clusters **(D)** Differential expression of TXNL4A in cell clusters of GSE140228_10X. **(E)** GSE166635 is identified as 21 cell clusters **(F)** Cell cluster annotation of GSE166635 **(G)** Distribution of TXNL4A abundance in immune cell clusters **(H)** TXNL4A expression in various cell clusters was not significantly different; however, it was predominantly highly expressed in immune cells.

The expression of TXNL4A in immune cells (GSE140228_10X and GSE166635) in HCC were analyzed using three scRNA cohorts in the TISCH2 database. In GSE140228_10X, TXNL4A was highly expressed in B, CD4, and CD8 cells (**Figures 7A–D**). In GSE166635, TXNL4A was highly expressed in CD8 T cells and monocytes/macrophages (**Figures 7E–H**). Previous immune infiltration analyses revealed a positive correlation between TXNL4A and the degree of infiltration of CD4 T cells, CD8 T cells, and DCs in HCC. Therefore, TXNL4A may be a potential factor influencing immune infiltration in HCC.

## Discussion

Patients with HCC have a high mortality rate because of disease heterogeneity and difficulty in early diagnosis. Furthermore, the metastasis, recurrence, and chemoresistance of HCC make the prognosis of patients with HCC poor (21–23). Therefore, a novel biomarker for HCC would be of great significance to patient health.

Imbalanced RNA splicing significantly threatens human health, often resulting in abnormal glucose metabolism, stroke, cardiovascular disease, and cancer (24). Targeting RNA shear factors is emerging as a potential antitumor therapeutic modality (25–27). Therefore, in this study, we screened HCC marker genes from the RNA splicing pathway. A novel marker gene for HCC, TXNL4A, was identified by constructing a prognostic model for RRGs. TXNL4A is a member of the RNA splicing body U5 snp and exerts oncogenic effects by affecting the homeostasis of RNA splicing (28). During carcinogenesis, the involvement of RNA splicing imbalances is necessary for changes in cellular metabolism, the achievement of growth factor independence, angiogenesis, and the onset of apoptotic resistance (29). TXNL4A expression in HCC, its effects on survival, enrichment analysis of positively and DEGs, and immunoassay results were comprehensively analyzed.

TXNL4A contributes to RNA splicing as a component of the U5 snRNP and U4/U6-U5 tri-snRNP complexes that are involved in spliceosome assembly, and also as a component of the precatalytic spliceosome (28, 30, 31). **Changes in RNA splicing are a source of functional diversity of proteins (32–34).The aberration of RNA splicing can affect HCC progression by altering E3 ubiquitin ligase activity, intercellular adhesion, and DNA methyltransferases (35–37). Abnormal RNA splicing has been reported to reduce cell death and thus affect HCC sensitivity to sorafenib (32). Immunity is primarily regulated by altering the efficiency of cytokine signaling (38).

We analyzed the effect of TXNL4A on survival, where patients with high TXNL4A expression experienced worse OS than those with low TXNL4A expression. The same results were replicated in the ICGC-HCC, GEPIA, and UALCAN databases. On analyzing the relationship between TXNL4A and the pathological HCC features, TXNL4A expression was found to be correlated with T-stage (p < 0.001), pathologic stage (p < 0.001), serum AFP level (p = 0.006), vascular invasion (p = 0.022), and mortality events. In this study, TXNL4A was identified as a prognostic biomarker for HCC and was closely correlated with T-stage, pathologic stage, and serum AFP level. However, TXNL4A did not correlate with N and M stages, which may be because lymph node and distant metastases are not the primary metastatic pathways in HCC (39). TXNL4A may be correlated with vascular invasion because the primary metastatic pathway in HCC is intrahepatic metastasis, and one of the mechanisms underlying metastasis is microvascular infiltration (40–43). Multiple regression analyses revealed that TXNL4A is an independent prognostic factor for HCC. Therefore, we believe that high TXNL4A expression is a potential factor in promoting HCC proliferation and worse OS.

Gene enrichment analyses can better indicate pathways where the genes may be located and allow the exploration of pathways *via* which they might function (44–46). As mentioned above, high TXNL4A expression is associated with the vascular invasion of HCC. The enrichment analyses also revealed similar results, and the genes in the group with high TXNL4A expression were enriched in tumor invasion-related pathways. Furthermore, GSEA revealed that genes in the group with high TXNL4A expression were highly enriched in pathways of stem cells and undifferentiated cancers. Therefore, this finding suggests that high TXNL4A expression is a risk factor for carcinogenesis.

HCC development has been reported to be highly correlated with tumor immune infiltration. Imbalanced RNA splicing generates recognizable neoantigens by altering the major histocompatibility complex I-binding immunopeptide, which triggers an antitumor immune response in T cells, including CD8 T cells, in the body (47). The RNA splicing pathway affects tumor immunity and is a potential target for tumor immunotherapy (12). TXNL4A is involved in the immune infiltration of pancreatic cancer; therefore, it was hypothesized that TXNL4A might influence the immune infiltration of HCC (15, 48, 49). A series of analyses and validations revealed that TXNL4A expression was positively correlated with levels of CD8 T cells. Targeting CD8 cells is a promising immunotherapeutic strategy for HCC; therefore, TXNL4A may serve as a novel therapeutic target (50–53).

In conclusion, we identified TXNL4A as a novel biomarker for HCC in the RNA splicing pathway for the first time. TXNL4A is highly expressed in HCC, suggesting that TXNL4A is an oncogene in HCC. Using survival analyses, high TXNL4A expression was found to be a potential risk factor for worse HCC prognosis. This study revealed using bioinformatic analyses that TXNL4A can be used as a novel biomarker for prognosis and immune correlation in HCC. Considering TXNL4A is a gene in the RNA splicing pathway, TXNL4A is a promising novel target for RNA shear-targeting drugs or RNA vaccines (24, 54, 55).

## Data availability statement

The original contributions presented in the study are included in the article/**Supplementary Material**. Further inquiries can be directed to the corresponding author.

## Author contributions

CQ contributed to the conception and design of this paper. YL, QZ and SZ downloaded and organized related papers. YL, QZ, JC and AD contributed to all tables and figures and the main manuscript. YL, QZ, and AD revised the manuscript. All authors contributed to the article and approved the submitted version.
